# Optimized DPPH assay in a detergent-based buffer system for measuring antioxidant activity of proteins

**DOI:** 10.1016/j.mex.2014.10.004

**Published:** 2014-10-22

**Authors:** Sascha C.T. Nicklisch, J. Herbert Waite

**Affiliations:** Marine Science Institute, Department of Molecular, Cell & Developmental Biology, University of California, Santa Barbara, CA 93106, USA

**Keywords:** DPPH radical, Antioxidant proteins, Non-ionic detergent, pH range

## Abstract

The free radical method using 1,1-diphenyl-2-picryl-hydrazyl (DPPH) is a well established assay for the *in vitro* determination of antioxidant activity in food and biological extracts. The standard DPPH assay uses methanol or ethanol as solvents, or buffered alcoholic solutions in a ratio of 40%/60% (buffer/alcohol, v/v) to keep the hydrophobic hydrazyl radical and phenolic test compounds soluble while offering sufficient buffering capacity at different pHs tested. Following this protocol, we were unable to keep proteinaceous antioxidants soluble at different pHs to test for their antioxidant activity. Thus, the assay protocol was modified as follows to improve its utility:

• Non-ionic detergents were added to keep the DPPH radical soluble and to provide a mild and non-denaturing environment for the antioxidant protein.
• Maximal concentration of DPPH was limited to 100 μM to stay within the sensitivity range of the detector at the given wavelength (515 nm) and to increase the dynamic range of the assay.
• 0.1 M citrate phosphate buffer was introduced to prevent experimental artifacts due to changing buffer compositions at different pHs.

## Method details

### Background information

A popular strategy to determine the antioxidant activity of a given compound *in vitro* is to directly measure the ability to scavenge specific free radicals. One approach is to monitor the reducing ability of antioxidants toward the commercially available stable DPPH radical [1]. In its radical form DPPH absorbs at 515 nm but upon reduction the absorption disappears and the color of the solution changes from violet to pale yellow [2]. Simplicity and speed of analysis of this substrate-free method has been shown for a broad range of mono- and polyphenolic compounds [3]. However, one of the major drawbacks of this method is that application of the conventional methanol and buffered methanol-based DPPH assay misses a large contribution from proteins and other hydrophilic antioxidants because these are precipitated by the solvent (refer to [4] for a comparison of buffer systems). In addition, seemingly higher DPPH radical scavenging activity of polyphenols reconstituted in protic solvents such as methanol as compared to aprotic solvents have been observed [3].

We developed a protocol that uses mild non-ionic detergents to keep both the DPPH radical and known antioxidant proteins soluble and stable in the assay buffer during a time course of up to 60 min. Our protocol is organized in four discrete (consecutive) steps that allow it to be tailored to different protein/detergent pairings. The steps are: (i) determining the minimum detergent concentration to keep the DPPH radical stable over time, (ii) determining the linearity of DPPH absorption at different detergent buffer pHs, (iii) testing DPPH radical scavenging by control antioxidants as internal standard in the detergent buffer, and finally (iv) determining DPPH radical scavenging by protein antioxidants in the detergent buffer.

## Determining minimum detergent concentration to keep DPPH radical stable

A final concentration of 0.3% (v/v) Triton X-100 (about 20 times the critical micelle concentration, cmc) was selected to keep 100 μM DPPH soluble and stable for up to 16 h (Fig. 1). If other detergents have to be investigated, we recommend first testing different detergent concentrations as shown in Fig. 1 and using the lowest concentration that shows long-term DPPH radical stability to prevent possible protein or peptide denaturation in the final assay.

### Procedure

• A 2 mM methanolic solution of DPPH was freshly prepared and wrapped in aluminum foil to prevent photochemical decomposition as mentioned in [5].
• 50 μl of the 2 mM DPPH stock solution was added to 950 μl detergent-based buffer to achieve a final DPPH concentration of 100 μM.
• The solution was mixed gently by pipetting half the volume (500 μl) 3–5 times.
• Each cuvette was sealed with Parafilm to prevent evaporation and incubated at RT under aluminum foil.
• After 5 min, 60 min, and 16 h of incubation, the A_515_ was determined in triplicate. Absorbance was measured in double distilled water (H_2_O), pure methanol (MeOH), and in water supplemented with different concentrations of the mild non-ionic detergents Tween 20 or Triton X-100 (Fig. 1).

## Determining linearity of DPPH absorption at different detergent buffer pHs

We determined that 100 μM DPPH reconstituted in 0.1 M citrate phosphate buffer supplemented with 0.3% (v/v) Triton X-100 at three different pHs is within the sensitivity range of the detector for the given wavelength (515 nm) and follows the Lambert–Beer law (Fig. 2). We recommend normalizing the DPPH absorbance to compare the kinetics at different pHs (see “Additional Information”).

### Procedure

• A 0.1 M solution of citrate phosphate buffer was prepared with final pHs of 3, 5, and 7.5 according to Ref. [6].
• 300 μl of a 10% (v/v) Triton X-100 solution was added to 9.7 ml of 0.1 M citrate phosphate buffer to achieve a final concentration of 0.3% (v/v) Triton X-100.
• 50 μl of a 0.5, 1, 2, 3, or 4 mM methanolic DPPH solution were added to 950 μl detergent-based buffer solution to achieve a final concentration of 25, 50, 100, 150 or 200 μM DPPH (Fig. 2A). Notably, there was no upshift in the pH upon the addition of the detergent and the methanolic DPPH solution to the citrate phosphate buffer.
• The final solution was mixed gently by pipetting half the volume (500 μl) 3–5 times.
• Absorbance at 515 nm of each DPPH concentration and buffer pH was determined in triplicate after 15 min (Fig. 2A) or after 5, 10, 15, 30, 45, and 60 min incubation at RT for 100 μM DPPH (Fig. 2B).

#### Testing DPPH scavenging by control antioxidants in detergent buffer

As internal standard and for comparison to published methods, we recommend to test the antioxidant activity of the control compounds ascorbic acid (vitamin C) and the water-soluble derivative of vitamin E (TROLOX, 6-hydroxy-2,5,7,8-tetramethylchroman-2-carboxylic acid) in the detergent-based buffer (Fig. 3A and B).

### Procedure

• 2.5, 5, 10, 15, and 20 μM of the control antioxidant compound were directly reconstituted in 950 μl assay buffer.
• The reaction was started by adding 50 μl of 2 mM freshly prepared methanolic DPPH solution to the buffer to achieve a final DPPH concentration of 100 μM.
• The solution was gently mixed by pipetting half the reaction volume (500 μl) 3–5 times. Note that a carry-over effect at higher ratios of antioxidant/DPPH can be observed using the same pipette tip in mixing the triplicate samples. This leads to higher standard deviations at higher antioxidant concentrations and needs to be considered during data analysis.
• The solution mix was incubated for a total of 120 min at RT and the absorbance at 515 nm of each sample was measured in triplicate after 5, 10, 15, 30, 45, 60, 75, 90, 105, and 120 min (Fig. 3).

## Determining DPPH scavenging by protein antioxidants in detergent buffer

For the time course experiment of DPPH radical quenching by proteinaceous antioxidants (Fig. 4) in the new detergent-based buffer we selected a recently identified antioxidant protein from *Mytilus californianus*, a recombinant version of this protein, as well as the two control proteins lysozyme and BSA known to have antioxidant properties [7–9].

### Procedure

• Five micromoles of pure protein was freeze-dried and dissolved in the assay buffer (0.1 M citrate phosphate buffer supplemented with 0.3% (v/v) Triton X-100) with a final volume of 950 μl.
• The cuvette with the protein/buffer mix was inserted into the spectrophotometer to measure within 1–2 min upon start of the reaction.
• The reaction was started by adding 50 μl of 2 mM freshly prepared methanolic DPPH solution to the buffer to achieve a final DPPH concentration of 100 μM.
• The solution was gently mixed by pipetting half the reaction volume (500 μl) 3–5 times.
• The absorption at 515 nm was determined at 2 (right after mixing), 5, 10, 15, 30, 45 and 60 min at RT in triplicate. All samples were kept in the dark at RT during the measurements.
• Prior to each time point measurement, the samples were centrifuged 1 min at 17,000 × *g* to check for precipitates. If precipitates were visible, the supernatant was transferred into a fresh cuvette and measured. Note that precipitation leads to an increase in turbidity (*i.e.* absorbance at 515 nm) and consequently to false negative results.

## Conclusions

Among the various *in vitro* methods to measure the activity of antioxidants in food and biological samples, the substrate-free DPPH assay has become quite popular due to its simplicity and speed of analysis (for reviews on antioxidant assays refer to [11–13]). However, to this date no single *in vitro* assay has yet been validated and approved by the scientific community as the “Swiss Army Knife” able to measure the total antioxidant activity or capacity of complex multiphase systems present in food or biological samples. Instead, a combination of test procedures with a careful selection of antioxidant assays based on the system under study and question to be addressed have been proposed [11,12]. We selected the substrate-free DPPH assay for our studies because its interaction kinetics with polyphenolic and non-phenolic compounds have been extensively characterized in the last two decades [2,3,14,15]. In our particular case, we were interested in the total antioxidant activity of a recently identified basic mussel foot protein (Mfp-6) from the California mussel (*M. californianus* [16]). Comparing the radical scavenging activity of the native cysteine- and tyrosine-rich (some of them converted to DOPA) Mfp-6 protein with a recombinant version lacking the DOPA residues [8] allowed us to obtain preliminary data on the effect of DOPA residues on the kinetics and total antioxidant activity of this extracellular acting natural antioxidant protein. Although a detailed kinetic analysis of the interactions of Mfp-6 and rMfp-6 with the DPPH radical is beyond the scope of this article, the authors believe that the optimized DPPH assay protocol described above (see section “Determining DPPH scavenging by protein antioxidants in detergent buffer”) could be a complementary, rapid and simple *in vitro* method to identify total antioxidant activity of soluble and possibly membrane proteins or peptides that would otherwise denature and precipitate in the conventional methanol and ethanol based buffer systems.

By using control antioxidants (ascorbic acid, TROLOX) as standards we were able to determine absolute parameters of antioxidant activity (EC_50_) that were identical to what was published with the standard ethanol- or methanol-based buffer system. For example, EC_50_ values of 0.24 (ascorbic acid) and 0.33 (TROLOX) mole antioxidant per mole DPPH radical were obtained from the fitted graphs in Fig. 3 and are in good agreement with the values for ascorbic acid obtained by [4] when the reaction was measured in methanol (0.24) or buffered methanol (0.23). The similar reactivity of TROLOX and ascorbic acid in terms of stoichiometry and kinetics toward the DPPH radical could also be observed by [17] in 60% ethanol/40% citrate buffer (10 mM, pH 3). We then calculated relative antioxidant activity values (VCEAC, TEAC) for the respective proteins to provide a second measure for total antioxidant activity of proteins relative to ascorbic acid and TROLOX. Preliminary data in our lab further indicated that thiol-containing compounds show a different kinetic with the DPPH radical than hydroxyl group-containing compounds using this new buffer system, potentially offering a convenient method for future studies to discriminate antioxidant-active residues involved in the total antioxidant activity of a given peptide, protein or protein mixtures under study [18].

## Additional information

### Time course of spontaneous reduction of DPPH radical in buffered methanol

As compared to the standard protocols [4], the control absorbance of, *e.g.* 50 μM DPPH in buffered methanol is not stable during the 60 min course in our study and steadily decreases over time (Fig. 5). In addition, we observed that mixing methanol and 0.1 M citrate phosphate buffer in a ratio of 60:40 (v/v) causes a pH upshift of about 1 units and readjustment of the pH is necessary for all three tested pHs (pHs 3, 5, and 7.5).

### Normalizing DPPH absorbance decrease in multi-buffer pH comparison

The time course of a protein's antioxidant activity at a given buffer pH has to be determined by plotting the DPPH absorbance reduction relative to the respective control value in percentage. This is necessary because the control absorbance of 100 μM DPPH has slightly different maxima, and probably different extinction coefficients, under the different buffer pHs (Fig. 2A and B). If this effect occurs due to a proton-transfer-mediated electron transfer [19] to the DPPH radical (p*K*_a_ = 8.6) at the low pH of the assay, or due to other possible radical quenching effects, needs to be addressed in future studies and lies beyond the scope of this study. However, a bathochromic shift effect of the Triton X-100 detergent at the absorption peak could be observed and is likely to contribute further to the lower relative absorption of 100 μM DPPH dissolved in the new buffer system at pH 3, 5, and 7.5 as compared to pure methanol (graphical abstract figure and [20]). In contrast to the standard assay protocols, a slight decrease (about 2–3%) in the control absorbance of 100 μM DPPH at prolonged time (>60 min) was observed in almost every experiment and is likely to be attributable to unavoidable DPPH radical precipitation, degradation or unidentified side reactions as mentioned in [3].

## Conflict of interest

The authors have declared no conflict of interest.

## Figures and Tables

**Fig. 1:**
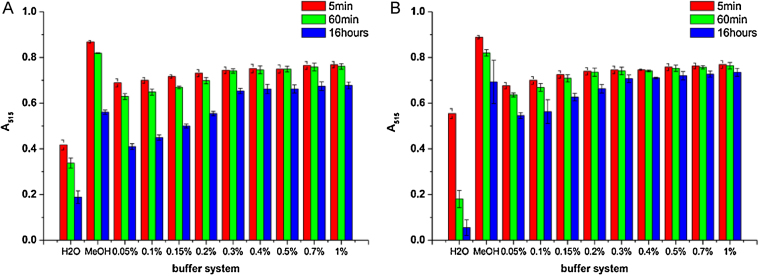
Optimization of the DPPH assay buffer for antioxidant proteins. Tween 20 (A) and Triton X-100 (B) concentrations are final concentrations in the buffer in v/v. Absorbance was measured in triplicates and after 5 min, 1 h and 16 h incubation. The data indicates the average A_515_ values ± SEM.

**Fig. 2:**
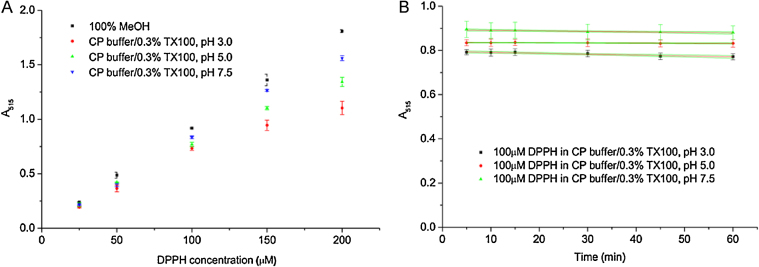
Linear relationship between DPPH concentration and absorbance in the new buffer system. 100 μM DPPH used for the experiments is within the sensitivity range of the detector for the given wavelength (515 nm) and follows the Lambert–Beer law (A). 100 μM DPPH radical shows minimal degradation in the new buffer system during the 60 min time course (B). The data were analyzed using a linear fit (*y* = *a* + b*x). Confidence bands are displayed with a 95% confidence level. All data points indicate the average values ± SEM from at least three measurements.

**Fig. 3:**
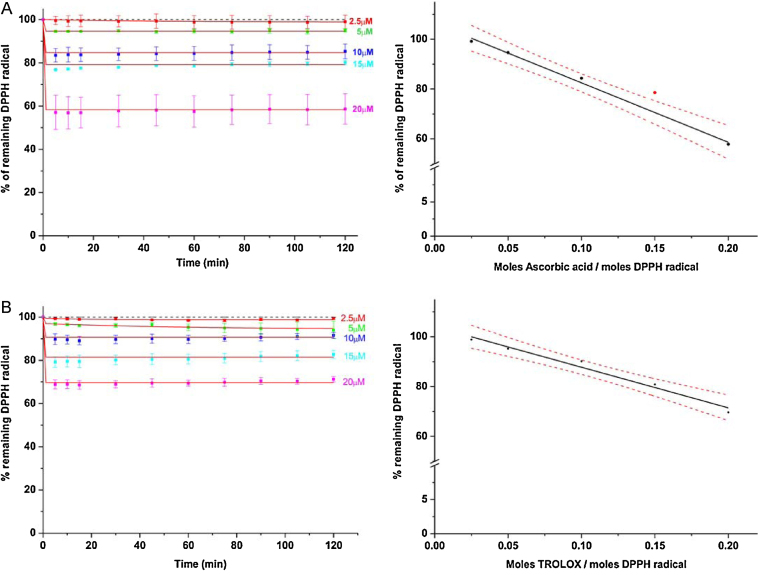
Time course of 100 μM DPPH radical reduction following addition of different concentrations of control antioxidants ascorbic acid and TROLOX at pH 3. The reaction mixture (1.0 ml) in 0.3% Triton X-100/0.1 M citrate phosphate buffer contained DPPH (100 μM) alone or in the presence of increasing concentrations of ascorbic acid (A) or TROLOX (B). For better comparison, each absorbance is displayed as percentage of the control absorbance with 100 μM DPPH in the respective buffer (short-dashed line). Data were fitted to an exponential decay using the function *y* = *A*_1_*exp(−*x*/*t*_1_) + *A*_2_*exp(−*x*/*t*_2_) + *y*_0_. Concentrations of the control antioxidants (in mole per mole DPPH radical) were plotted against the percentage of remaining DPPH radical after 30 min reaction time and extrapolated to obtain the EC_50_ value of ascorbic acid and TROLOX in the new buffer system (right panel in A and B). EC_50_ values were calculated from the fitted curve and represent the molar ratio of antioxidant to DPPH radical needed to decrease the initial DPPH concentration by 50%. Analysis of lower and upper confidence levels (95%) and linear or exponential correlation tests were determined using Origin 8.

**Fig. 4:**
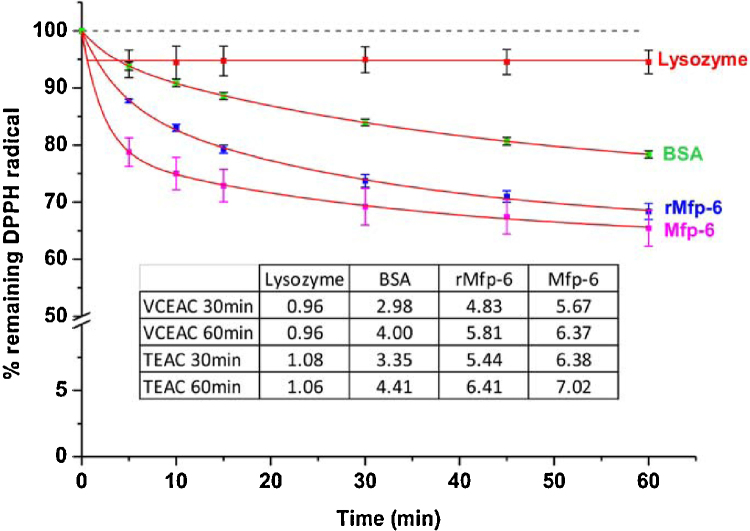
Time course of 100 μM DPPH radical reduction following addition of 5 μM antioxidant proteins: Mfp-6 [9], rMfp-6 [8], chicken egg white lysozyme and BSA in citrate phosphate buffer supplemented with 0.3% (v/v) Triton X-100 at pH 3.0. All data points indicate the average percentage of remaining DPPH radical ± SEM from at least three measurements. Data were fitted to an exponential decay using the function *y* = *A*_1_*exp(−*x*/*t*_1_) + *A*_2_*exp(−*x*/*t*_2_) + *y*_0_. The inset shows a table of the calculated VCEAC (vitamin C equivalent antioxidant capacity [10]) and TEAC (Trolox equivalent antioxidant capacity [11]) values for each protein antioxidant after 30 and 60 min reaction time.

**Fig. 5:**
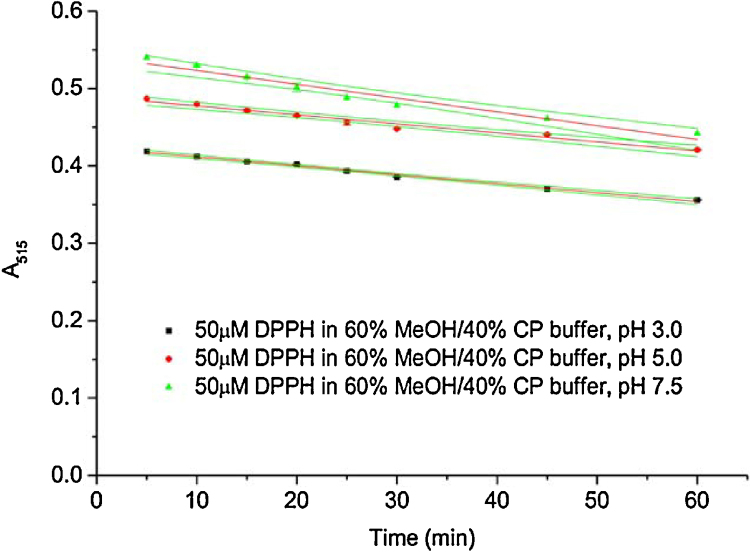
Time course of 50 μM DPPH radical decay in buffered methanol (60% methanol/40% citrate phosphate buffer, v/v). The decrease in absorbance was followed at 515 nm. MeOH = methanol, CP = citrate phosphate.

## Appendix A Supplementary data

Further information on EC_50_ values for the control antioxidant ascorbic acid at pH 5 and 7.5.
