# Supplementary material for: Optimized DPPH assay in a detergent-based buffer system for measuring antioxidant activity of proteins
Source: MethodsX. 2014 Oct 22;1:233–8. doi: 10.1016/j.mex.2014.10.004 (PMC4268772; doi:10.1016/j.mex.2014.10.004)
Supplement: Supplementary file 1 [file mmc1.docx]

*Supplementary Material*

We further determined the EC_50_ values for the control antioxidant ascorbic acid at pH 5 and 7.5 in the new buffer system to be able to apply the modified DPPH assay to neutral or more acidic antioxidant proteins and compounds (Figure S1 and S2). For better comparison, we determined all EC_50_ values for ascorbic acid after 30 min reaction time (Figure 3A in main manuscript and S1) according to the recommended reaction time mentioned in [1]. In addition, we calculated the EC_50_ values for the three pHs after 10, 30, 60, 90, and 120 min to emphasize the importance of discriminating EC_50_ values measured during the initial phase of the reaction and at prolonged reaction time according to [2]. A certain pH dependence of the reaction is obvious and has already been broadly discussed in literature for the reaction of polyphenols with the DPPH radical [3, 4, 5]. The characteristic biphasic decay of the DPPH radical with a fast step followed by a much slower rate of consumption is evident in Figures 3 (main manuscript) and Figure S1 and could also be observed for ascorbic acid, TROLOX and other polyphenolic compounds [1, 6, 7].

Figure S1: Time course of 100 μM DPPH radical reduction by different amounts of ascorbic acid (2.5-20 μM) in citrate phosphate buffer supplemented with 0.3% v/v Triton X-100 at pH 5.0 (A) and pH 7.5 (B). All data points indicate the average percentage of remaining DPPH radical ±SEM from at least three measurements. Concentrations of ascorbic acid (in mole per mole DPPH radical) were plotted against the percentage of remaining DPPH radical after 30min reaction time and extrapolated to obtain the EC_50_ values at pH 5 and 7.5 (right panel in A and B). The EC_50_ values were calculated from the fitted curve and represent the molar ratio of antioxidant to DPPH radical needed to decrease the initial DPPH concentration by 50%. Analysis of lower and upper confidence levels (95%) and linear or exponential correlation tests were determined using Origin 8.

Figure S2: EC_50_ values for ascorbic acid in the new buffer system at pH 3, 5, and 7.5 as a function of the reaction time.

*References*

[1] Sharma, O., and Bhat, T. (2009) DPPH antioxidant assay revisited. *Food Chem* *113*, 1202–1205.
[2] Campos, A. M., Duran, N., Lopez-Alarcon, C., Lissi, E. (2012) Kinetic and stoichiometric evaluation of free radicals scavengers activities based on diphenyl-picryl hydrazyyl (DPPH) consumption. *J Chil Chem Soc 57(4)*, 1381–1384.
[3] Foti, M. C., Daquino, C., Geraci, C. (2004) Electron-transfer reaction of cinnamic acids and their methyl esters with the DPPH radical in alcoholic solutions. *J Org Chem* *69*(7), 2309–2314.
[4] Takebayashi, J., Tai, A., Gohda, E., Yamamoto, I. (2006) Characterization of the radical-scavenging reaction of 2-O-substituted ascorbic acid derivatives, AA-2G, AA-2P, and AA-2S: a kinetic and stoichiometric study. *Biol Pharm Bull 29(4)*, 766–771.
[5] Milardović, S., Iveković, D., & Grabarić, B. (2006) A novel amperometric method for antioxidant activity determination using DPPH free radical. *Bioelectrochemistry 68(2)*, 175–180.
[6] Sánchez‐Moreno, C., Larrauri, J. A., Saura‐Calixto, F. (1998) A procedure to measure the antiradical efficiency of polyphenols. *J Sci Food Agr* *76*(2), 270–276.
[7] Villano, D., and Fernández-Pachón, M. (2007) Radical scavenging ability of polyphenolic compounds towards DPPH free radical. *Talanta* *71*, 230–5.
